# Preparation of Volborthite by a Facile Synthetic Chemical Solvent Extraction Method

**DOI:** 10.3390/nano13131977

**Published:** 2023-06-29

**Authors:** María Guadalupe Sánchez-Loredo, Salvador Antonio Palomares-Sánchez, Gladis Judith Labrada-Delgado, Toni Helbig, Paul Chekhonin, Doreen Ebert, Robert Möckel, Jones Owusu Afriyie, Norman Kelly

**Affiliations:** 1 Helmholtz-Zentrum Dresden-Rossendorf e.V., Helmholtz-Institut Freiberg für Ressourcentechnologie, Chemnitzer Str. 40, 09599 Freiberg, Germany; tonihelbig@yahoo.de (T.H.); d.ebert@hzdr.de (D.E.); r.moeckel@hzdr.de (R.M.); n.kelly@hzdr.de (N.K.); 2 Instituto de Metalurgia, Facultad de Ingeniería, Universidad Autónoma de San Luis Potosí, Sierra Leona 550, San Luis Potosí 78210, Mexico; 3 Facultad de Ciencias, Universidad Autónoma de San Luis Potosí, Av. Chapultepec 1570, San Luis Potosí 78295, Mexico; sapasa04@fciencias.uaslp.mx; 4 Instituto Potosino de Investigación Científica y Tecnológica, Camino a la Presa San José 2055, San Luis Potosí 78216, Mexico; gladis.labrada@ipicyt.edu.mx; 5 Helmholtz-Zentrum Dresden-Rossendorf e.V., Institut für Ressourcenökologie, Bautzner Landstraße 400, 01328 Dresden, Germany; p.chekhonin@hzdr.de

**Keywords:** vanadium(V) extraction, anion exchange, quaternary ammonium salt, precipitation stripping, nanostructured vanadates, volborthite, polyvinylpyrrolidone

## Abstract

In this work, the extraction of vanadium (V) ions from an alkaline solution using a commercial quaternary ammonium salt and the production of metal vanadates through precipitation stripping were carried out. The crystallization of copper vanadates from the extracts was performed using a solution containing a copper(II) source in concentrated chloride media as a stripping agent. In an attempt to control growth, a stabilizing polymer (polyvinylpyrrolidone, PVP) was added to the stripping solution. The structural characteristics of the crystallized products, mainly copper pyrovanadate (volborthite, Cu_3_V_2_O_7_(OH)_2_·(H_2_O)_2_) nanoflakes and nanoflowers and the experimental parameter influencing the efficiency of the stripping process were studied. From the results, the synthesis of nanostructured vanadates is a simple and versatile method for the fabrication of valuable three-dimensional structures providing abundant active zones for energy and catalytic applications.

## 1. Introduction

In the last few years, mainly due to their high theoretical capacity and easy preparation and safety, transition metal vanadates have shown potential as materials for electrodes in primary and rechargeable lithium-ion batteries and as potential materials for sensors, phosphors, as well as for the photocatalytic treatment of organic pollutants and water splitting [1,2,3,4,5,6]. In this sense, efforts have been carried out on the synthesis and characterization of several metallic vanadates, mostly by means of energy- and time-consuming methods [1,2,7,8,9,10,11,12].

Particularly copper vanadates (CVO) have attracted great interest as semiconductor photocatalytic materials owing to their narrow bandgap that absorbs solar visible light [13]. They possess high-temperature stability, high voltage and capacity and have been proposed as potential cathodes in thermal batteries [12]. Copper vanadates have also been proposed as promising battery materials for primary or secondary lithium battery applications owing to their layered nature, multistep reduction reaction and excellent kinetics during the insertion/intercalation of lithium [14]. During the discharge process, Cu^2+^ is reduced to Cu^0^ and more than one Li^+^ per vanadium can be inserted.

Different types of mixed oxides based on copper and vanadium, among them CuV_2_O_6_, Cu_2_V_2_O_7_, Cu_3_V_2_O_8_ and Cu_3_(OH)_2_V_2_O_7_·nH_2_O, have been synthesized and characterized [12,13,15,16,17,18,19,20,21,22,23]. Normally, hydrothermal synthesis is proposed [13,21,22,23], where the use of special equipment (autoclaves), long reaction times and high temperatures is required, making the process less practical, energy-intensive, expensive and difficult to scale up.

During a typical hydrometallurgical process including chemical leaching, one of the more efficient procedures for the separation and purification of valuable metallic species is the solvent extraction process. Solvent extraction usually consists of extraction, scrubbing and stripping. In this last step, the solute is returned from the organic to an aqueous phase for recovery, for instance by electrowinning. An interesting modification of the conventional hydrometallurgical process is the addition of a crystallization operation directly from the organic phase, where low-solubility metal salts such as oxides or sulfides can be precipitated (precipitation stripping) [24,25,26,27,28]. As stated by Doyle some years ago [29], the solvent extraction systems provide convenient “inexpensively prepared, non-aqueous media in which precipitation can be controlled”. In addition, because the organic phase has a low dielectric constant and is free from ionic species, undesirable contamination of the products can be largely avoided.

A nanoparticle is a fundamental component in the fabrication of advanced materials, as nanometrical particles have different physical and chemical properties compared to bulk material. Several approaches can be used to prepare dispersed nanoparticles. Among them, precipitation from aqueous or nonaqueous media is commonly used because it is easy, cheap and versatile, offering many possibilities to control the particle characteristics (size, morphology) by changing the experimental parameters. However, in order to obtain highly pure materials powders, the starting reagents must be purified using separation techniques. The solvent extraction systems could thus become convenient media from which a controlled nanoparticle synthesis can be performed.

Therefore, as precipitation from organic media is an excellent and easy chemical route for preparing nanocrystalline materials and can be easily performed using the organic phases from the solvent extraction process, in this work, the crystallization of metal vanadates from organic extracts at room temperature was carried out. The production of metal vanadates from organic phases from solvent extraction has, until now, as far as we know, not been reported in the literature.

In order to achieve this, organic extracts were obtained by mixing V(V) from an 0.1 M NaOH solution with a mixture of Aliquat^®^336 (methyltrialkylammonium chloride)/1-octanol/kerosene. The precipitation stripping was performed by adding to the organic phase, while stirring and at a constant rate, a stripping solution composed of Cu(II) ions in a concentrated chloride solution. The obtained materials were separated from the organic solution, washed and characterized chemically, structurally and microscopically. Different parameters affecting the recovery of V(V) and the composition of the vanadates, as well as their morphological characteristics, were studied.

## 2. Materials and Methods

### 2.1. Materials and Experiments

The chemical synthesis was performed by extracting the V(V) ions (prepared as a 2 g/L solution using V_2_O_5_ and 0.1 M NaOH) with an organic phase composed of Aliquat^®^336 (10 or 20%)/1-octanol (5 or 10%)/kerosene. The extraction time was 1 h for all the experiments and the organic/aqueous phase ratio was 1:1, unless otherwise stated. The extraction rate was determined by analyzing the aqueous phase before and after extraction by means of inductively coupled plasma optical emission spectrometry (ICP-OES).

Instead of using the conventional stripping, the nonconventional alternative called precipitation or crystallization stripping was used, where the metal ions, V(V), in the loaded organic phase, can be directly recovered as vanadate powders using Cu(II) in highly concentrated chloride solutions. The first set of precipitation stripping experiments (PS-1, PS-2 and PS-3) was carried out by adding 100 mL aqueous strip solution at a constant rate to 100 mL V(V)-loaded organic solution (total addition time 10 min). CuSO_4_ concentration was 0.05 or 0.1 mol/L and the chloride concentration was 4 mol/L. In experiment PS-3, the stabilizing polymer PVP was added to the strip solution in order to try to influence growth and thus prepare smaller and nonaggregated particles. Another experiment (CP-1) was performed where the metal sulfate was dissolved in the 4 M chloride solution and 20 g/L PVP (Cu(II) concentration 0.05 mol/L) and mixed with the same volume of a 2 g/L V(V) solution in 0.1 M NaOH, avoiding the extraction step. The experimental conditions of this first set (Set 1) of experiments are presented in Table 1.

The mixing time after finishing addition was 1 h and the powders were recovered through centrifugation and washed several times with water, water/ethanol mixtures (50:50 *v*/*v*) and, finally, acetone. The powders were dried in a vacuum oven at room temperature and characterized by means of X-ray diffraction (XRD), X-ray fluorescence (XRF), scanning electron microscopy (SEM) and infrared spectroscopy (FTIR).

To get a better understanding of the growth process and with the aim of optimizing the stripping procedure, a second set of experiments (Set 2) was carried out, varying several experimental parameters (Table 1).

### 2.2. Methods

Bulk chemical assays were carried out using inductively coupled plasma optical emission spectrometry (ICP-OES, PlasmaQuant PQ9000 Analytik Jena GmbH+Co, Jena, Germany) and portable X-ray fluorescent spectrometry (pXRF, Bruker S1 Titan). X-ray diffractograms (XRD) of the samples were obtained with an Empyrean diffractometer (PANalytical, Almelo, The Netherlands) equipped with a PIXcel3D-Medipix area detector (in combination with a Fe-filter) and a proportional counter (with monochromator) as well as a Co X-ray tube (Kα1 = 1.789010 Å) anode and a voltage and current of 35 KV of 35 mA, respectively. The scan range was 5°–80° with a step size of 0.0131° and an overall measurement time of 10 h. The irradiated area was kept constant (10–12 mm) by means of an automated divergence slit. The Rietveld refinement method of the structure was used to analyze the structural parameters of the phases that constitute the samples. This is also a method for the quantitative determination of the phases present in a compound and/or mixture of amorphous and crystalline phases [30]. The MAUD program was used for the refinement analysis [31]. For the second set of experiments, quantifications were implemented utilizing the Rietveld method using the BGMN/Profex software package v. 5.0 [32,33].

Samples for scanning electron microscopy (SEM) were prepared by solving small amounts of nanoparticle powders in alcohol. Subsequently, the particle solutions were evaporated on aluminum stubs acting as SEM sample holders. SEM micrographs were recorded using secondary electron (SE) imaging in an EVO 50 SEM (Zeiss), located at HZDR, Rossendorf, equipped with a tungsten filament, which was operated at 15 kV acceleration voltage.

Energy dispersive X-ray spectroscopy measurements were performed as point measurements using a Bruker EDX system. In order to ensure smooth EDX spectra with reliable statistics, the integration time for each point measurement was set to 3 min. Because the particles or the agglomerations of those were typically below 1 µm in size, the electron beam would penetrate them and, consequently, the aluminum sample holder would give a contribution to the EDX spectrum. In order to keep this contribution as small as possible, larger particle agglomerations were chosen as spots for EDX measurements if a quantitative evaluation was of interest. In such cases, possible traces of an aluminum peak in the spectra were ignored during the quantitative evaluation.

In addition, a small number of selected samples was dispersed in isopropanol and subjected to sonication for 10 min; after that, an aliquot was poured onto a copper grid, vacuum-dried and, after that, images using both backscattering and secondary electron detectors were obtained using field emission gun scanning electron microscopy (FEG-SEM) with a FIB Dualbeam FEI Helios 600 Nanolab Scanning Electron Microscope (Hillsboro, FL, U.S.A.), located at IPICyT (San Luis Potosí, Mexico). Infrared spectra were obtained using a portable Cary 630 FTIR spectrometer (Agilent, Santa Clara, CA, USA) with ATR.

## 3. Results and Discussion

A vanadium extraction rate of 76.6% was obtained with the organic system consisting of Aliquat^®^336 20%(*v/v*)/1-octanol 10%(*v/v*)/kerosene. This corresponds to an organic phase loaded with 1.52 g/L V(V), which was separated by centrifugation from the aqueous phase. Later, the organic solution was equilibrated with CuSO_4_-NaCl solutions (experiments PS-1–PS-3) to perform the precipitation stripping.

The stripping solution was added to an aliquot of the organic phase while stirring. The chloride ions act as the strip species by displacing the vanadate ions from the organic solution and the Cu(II) ions act as the precipitation agent, forming the sparingly soluble copper vanadates as finely dispersed powders, located mostly in the aqueous phase or at the interphase. Centrifugation of the mixture allowed the separation of the obtained oily powders and both liquid phases. The excess of organic and metal salts was removed from the powders by washing.

The structure of the copper vanadates PS-1 and PS-2 was determined by means of XRD and the chemical composition was obtained by XRF. The broad diffraction peaks (Figure S1, Supplementary Information File) could be indexed to the Cu_3_V_2_O_7_(OH)_2_·2H_2_O phase, space group: *C*2/*m*(12), using the JCPDS Card No. 46-1443 with the monoclinic structure. Apparently, only a single vanadate was obtained (a small amount of a chloride salt is present, but can be removed by further washing). The broad peaks indicate that the powders either have a not-well-defined structure or that the crystallite size is nanometrical.

**Figure 1:**
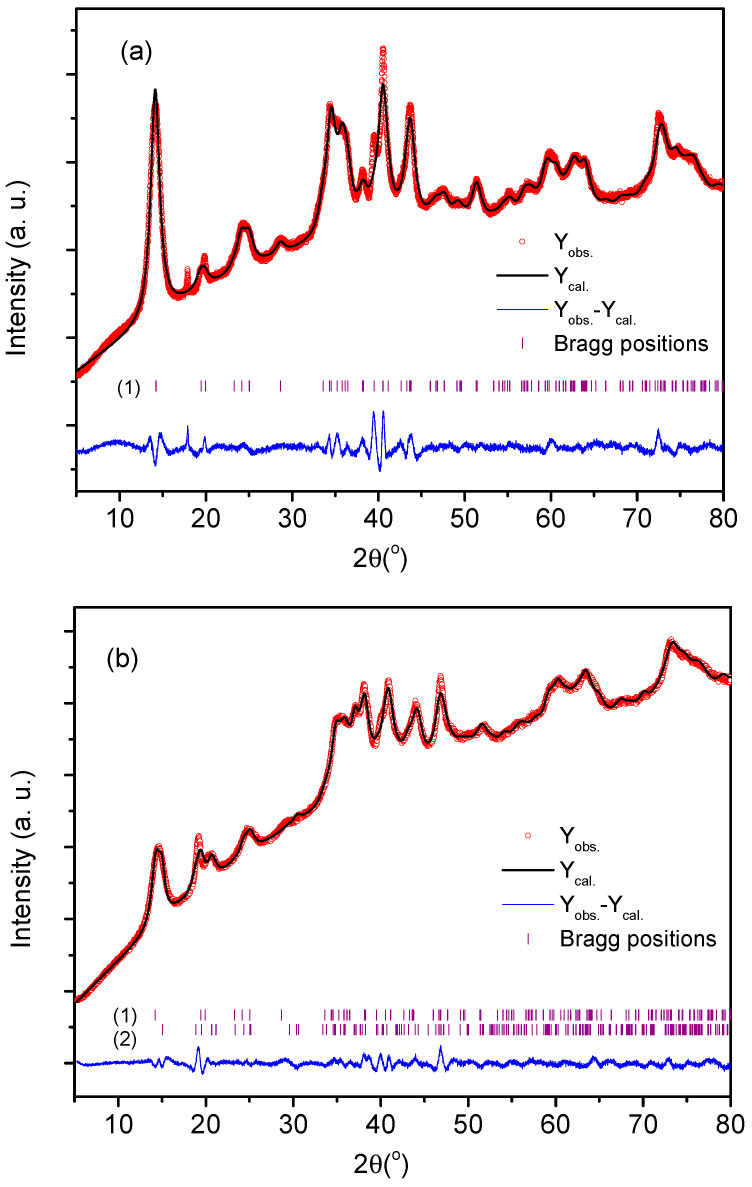
Observed (red) and calculated (black) X-ray diffractograms of samples PS-1 (**a**) and PS-2 (**b**). The vertical bars indicate the angular positions of the allowed Bragg reflections where (1) corresponds to Cu_3_V_2_O_7_(OH)_2_·2(H_2_O), space group *C*2/*m* and (2) corresponds to Cu_3_V_2_O_7_(OH)_2_·2(H_2_O), space group *C*2/*c*.

For the Rietveld refinement and as initial models, the following phases were used: Cu_3_V_2_O_7_(OH)_2_·2(H_2_O), monoclinic, space group C2/m [34]; Cu_3_V_2_O_7_(OH)_2_·2(H_2_O), monoclinic, space group *C*2/*c* [35]; Cu_5_V_2_O_10_, monoclinic, space group *P*2_1_/*c* [36], Cu_3_V_2_O_8_(H_2_O), monoclinic, space group *P*2_1_/*m*; and CuV_2_O_6_(H_2_O)_2_, monoclinic, space group *P*2/*c* [37]. The strategy of refinement was as follows: Initially, all the phases were used as models for the refinement. Afterward, only the adjusted phases were left for the final refinement of every compound. The results are shown in Table 2 and the refined diffractograms in Figure 1. Refinements are acceptable, considering that the contribution of the diffractometer to the width of the peak is not known and also that the particles are nanometrical in size or of amorphous nature. Diffraction peaks were again indexed to the monoclinic phase of Cu_3_(OH)_2_V_2_O_7_·2H_2_O. The results of the crystallite size correspond to monocrystals and, possibly, the particles are larger. Sample PS-3 could not be refined, as fluorescence makes the resolution low. Ghiyasiyan-Arani and col. [38] reported for a copper pyrovanadate nanostructure the same *C*2/*m* group and similar cell constants (*a* = 10.6060 Å, *b* = 5.8740 Å and *c* = 7.2130 Å, JCPDS Card No. 80–1169).

By comparing the particle size distribution of the products from experiments PS-1 and PS-2, where copper(II) concentration in the strip solutions was varied, a lower metal concentration led to a slightly smaller crystallite size. If it is considered that the copper(II) ions act as the precipitating reagent (chloride concentration remains the same, so that at the beginning the same quantity of V(V) goes to the aqueous phase), it can be assumed that more Cu(II) ions affect the solubility equilibrium and this could influence the size of the resultant particles.

Aliquat^®^336 (methyltrialkylammonium chloride), is a liquid anion exchanger based on short-chain quaternary ammonium cations and an effective phase transfer catalyst and well-known metal extraction reagent. The use of Aliquat^®^336 as a capping agent replacing cetyltrimethylammonium chloride (CTAC) during the synthesis of metallic nanoparticles was reported by Naz and col. [39], where, apart from its nontoxic nature, Aliquat^®^336 plays the same role as CTAC in the synthesis of highly faceted Au nanoparticles. Previous studies indicated twinning and preferential binding of CTAB (cetyltrimethylammonium bromide) molecules on the (100) crystal face of gold particles as a mechanism for nanorod synthesis and growth [40].

**Figure 2:**
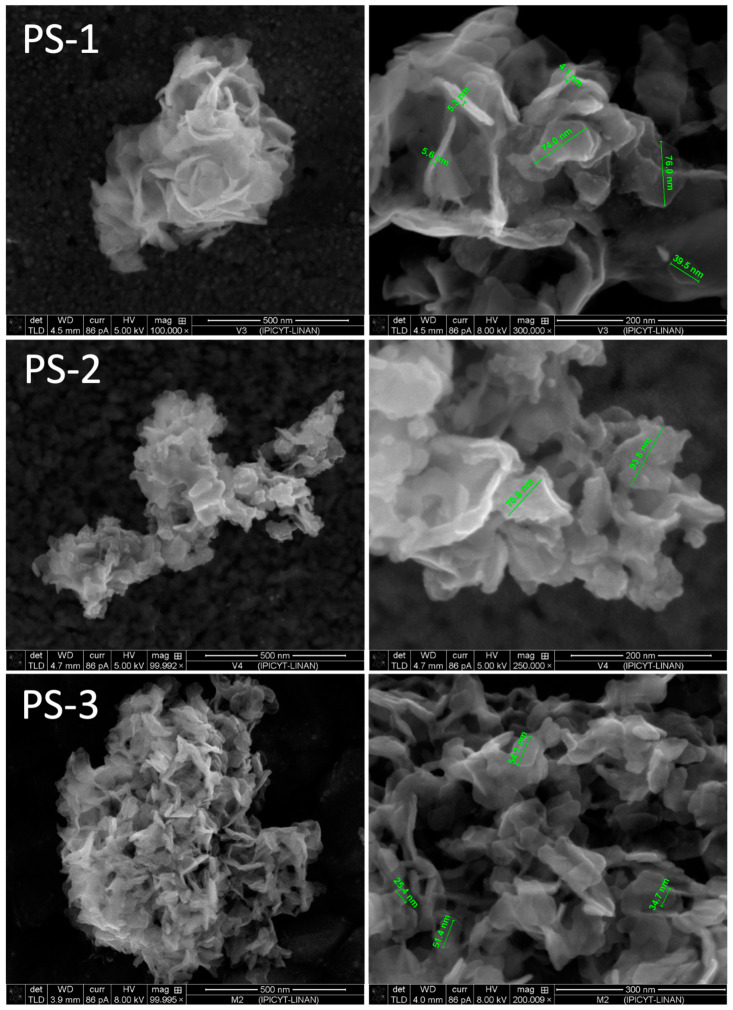
FESEM images of powders (PS-1, PS-2 and PS-3) obtained by precipitation stripping using solutions: PS-1 0.05 M Cu(II), PS-2 0.1 M Cu(II) and PS-3 0.01 M CuSO_4_, 20 g/L PVP in 4 M NaCl.

Taking into account the advantages of using Aliquat^®^336 as an extractant and a surfactant-directing agent, it was hoped that the reagent would help to synthesize a variety of vanadate-based nanostructures. The field emission SEM images of the prepared materials are shown in Figure 2. Micrographs of PS-1, PS-2 and PS-3 samples show hierarchically structured copper vanadate-flake-like particles. The thickness of the two-dimensional nanoflakes does not seem to vary greatly and lies in the range of about 4 to 8 nm.

The average diameter of the nanoflakes is 60 nm. Nevertheless, PS-2 and PS-3 show important aggregation and the morphology is less regular than in the case of PS-1. Interestingly, PS-1 appears to be composed of self-assembled ultra-thin sheets of 3D nanopetals, curved and with exposed sharp edges. Three-dimensional architectures formed from the assembly of two-dimensional nanoflakes are interesting due to the unique properties of their building blocks, providing exposed electroactive sites for catalysis, as well as charge storage [41]. This kind of three-dimensional structure provides superior electronic transport and ion transfer channels and an important increase in the contact area between the material and the electrolyte, thus achieving improved electrochemical reaction kinetics [42]. The synthesis of vanadate nanoflakes was already reported by Mahmoud and col. [43]: they obtained zinc vanadate as a highly porous structure in the range of 50 nm wall thickness using a simple coprecipitation method.

The energy dispersive X-ray spectrum (EDX) of nanostructures belonging to sample PS-3 is presented in Figure S2 and confirms the presence of Cu, V and O in the sample. It must be taken into account that the chemical analysis considers also additional copper signals induced by the copper grid, as well as signals from other elements from the sample holder.

**Figure 3:**
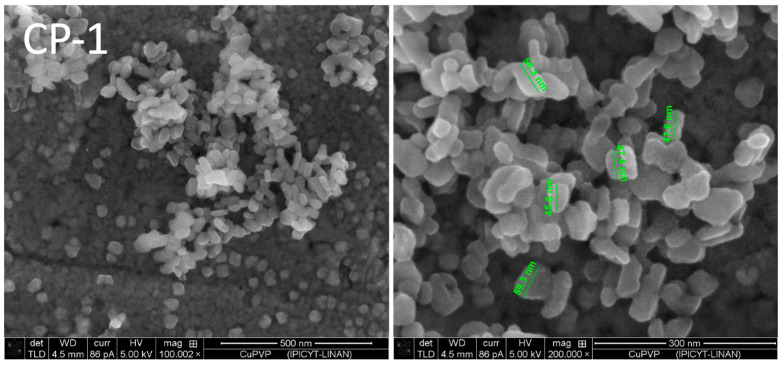
FESEM images of particles obtained by direct precipitation (Cu(II) 0.05 M, with 20 g/L PVP), in contact with V(V) aqueous solution (2 g/L in 0.1 N NaOH).

As a comparison, some preliminary experiments of precipitation from the aqueous phase were carried out in the absence and in the presence of PVP in order to find out if the polymer had in a homogeneous system a more important role as a nucleation and crystal-growth assistant. As an example, an experiment was carried out where the Cu(II)- and PVP-containing solution was added to an aqueous alkaline vanadate solution. The resulting particles (Sample CP-1, Figure 3) are well dispersed and show a rod-like morphology; similar particles, but of micrometrical sizes, were prepared by the sol-gel method [16], corresponding to a copper vanadate of structure CuV_2_O_6_. The experiment CP-1 leads to nanometrical single particles different from those obtained from the organic solutions; the stabilizer PVP seems to take a more important role in controlling size and growth in aqueous media.

The FESEM characterization shows that precipitation stripping is a facile and appropriated method to obtain ultrafine particles of interesting morphologies. According to these observations, the possibility of different copper–organic interactions during the precipitation stripping must be assumed. The detachment of vanadate ions from the organic extractant (Aliquat^®^336) through replacement with chloride ions, present in high concentration in the aqueous stripping phase, leads to locally low concentrations of vanadate ions at the phase boundary between the aqueous and the organic phase. This leads to the formation of a certain amount of nuclei of the sparingly soluble copper vanadates, where the precipitation equilibrium depends greatly on the amount of Cu(II) ions. The growth step leads to nanostructures different from that formed in the bulk of an aqueous solution, such as the ones presented in Figure 3.

For this reason, in order to find out if the particles are covered by an organic layer acting as a stabilizer and/or as a growth-control reagent, ATR measurements were carried out at the solid samples PS-1 and PS-3 (Figure S3) and the spectra were compared to the ones of Aliquat^®^336 and PVP (Figure S4). Infrared spectroscopy is a useful technique for studying the interaction of organics with solid surfaces. The strong bands featured in the Aliquat^®^336 spectrum (Figure S4), centered at 2852 cm^−1^ and 2921 cm^−1^ (corresponding to symmetric and asymmetric sp^3^ C-H stretching) and 1465 cm^−1^ (CH_2_-bending) [39], are absent in the IR spectra of the metal vanadates in Figure S3. Furthermore, the lack of the characteristic absorption band at 1377 cm^−1^ (CH_3_ bending) also indicates the absence of the extractant on the surface of the particles. The peak at 722 cm^−1^ in Figure S4 corresponds to the in-plane rocking mode of the methylene (-CH_2_-)_n_ chain of Aliquat^®^336, and the band at 1465 cm^−1^ was proposed to belong to (CH_3_)-N^+^ [44,45]. Nevertheless, there are signals corresponding to the polymer PVP in sample PS-3. The main IR vibrational assignments of PVP are (Figure S4): 3264 cm^−1^ -OH asymmetric stretching; 1635 cm^−1^, C=O stretching vibration; 1464 cm^−1^ CH_2_-bending and C-N stretching at 1294 cm^−1^ [46].

Bayat and col. [23] reported characteristic absorption peaks for colloidal volborthite, where the bands at 841 cm^−1^ and 1018 cm^−1^ are assigned to V-O-V and V-O vibrations, respectively. In this work, spectra of PS-1 and PS-3 show peaks at 832 cm^−1^ for PS-1 and 833 cm^−1^ for PS-3. The band at 744 cm^−1^ (PS-3) and 745 cm^−1^ (PS-1) can be assigned to the asymmetric vibrations of V-O-Cu and V-O-V [23]. Strong absorption peaks at 3545 cm^−1^ and 1616 cm^−1^ in the PS-3 spectrum were ascribed to the symmetric stretching and bending vibrations of H-O-H in H_2_O molecules, respectively [4]. The presence of PVP in PS-3 and the apparent absence of Aliquat^®^336 on the surface of the particles for both samples, added to the similar morphology, led to the assumption of the important role played by Aliquat^®^336 during particle growth controlling the release rate of V(V) ions from the organic phase, but the lack of affinity of the extractant toward the synthesized materials. The presence of PVP on the surface of PS-3 can be explained by the more hydrophilic character of the vanadates.

Table 3 shows the recovery rates of V(V) for the experiments PS-1, PS-2 and PS-3 (Set 1), where low yields of solids and distribution of V(V) among the solid and the organic phase were observed.

In an attempt to optimize the process and increase the recovery of V(V) as a solid, another set of parameters (Set 2, Table 1) was applied, where lower Aliquat^®^336 (10 % *v*/*v*), chloride (2 mol/L) and copper (0.05 mol/L) concentrations were selected. Some of the stripping solutions contained the stabilizer PVP in concentrations of 20 and 40 g/L. The results of the mass balances are presented in Table 4, and Table 5 presents the vanadium, copper and chloride percentage in the solids obtained for this set of experiments.

From these results, some conclusions can be pointed out. First, longer addition times (Set 2, 1 h instead of 10 min addition time) allow in general higher V(V) recoveries as a solid material; kinetics factors could then be affecting the solid precipitation rate. Other important parameters are the chloride concentration, where the lower anion amount leads to higher recovery and, very importantly, the presence of PVP, where 20 g/L is enough to achieve recoveries higher than 60%.

The V/Cu ratios in experiments PS-5, PS-6 and PS-7 are the closest to the theoretical V/Cu ratio expected for volborthite (0.53), but the presence of some amounts of chloride ions could correspond to the formation of some copper chlorides, difficult to eliminate during washing due to the fine nature of the material.

Table S1 shows the probable composition of the samples PS-4 to PS-11 obtained after treatment using Profex. Diffraction peaks in all samples could be indexed to the Cu_3_V_2_O_7_(OH)_2_·2H_2_O phase with the monoclinic structure (space group: *C*1 2/*m* 1, ICSD_63282), but the broadening of the peaks makes it difficult to refine the obtained diffractograms. No peaks from other vanadate phases have been detected; chlorides are present in some samples, but further washing of the samples could help to ensure the high purity of the nanostructured material.

**Figure 4:**
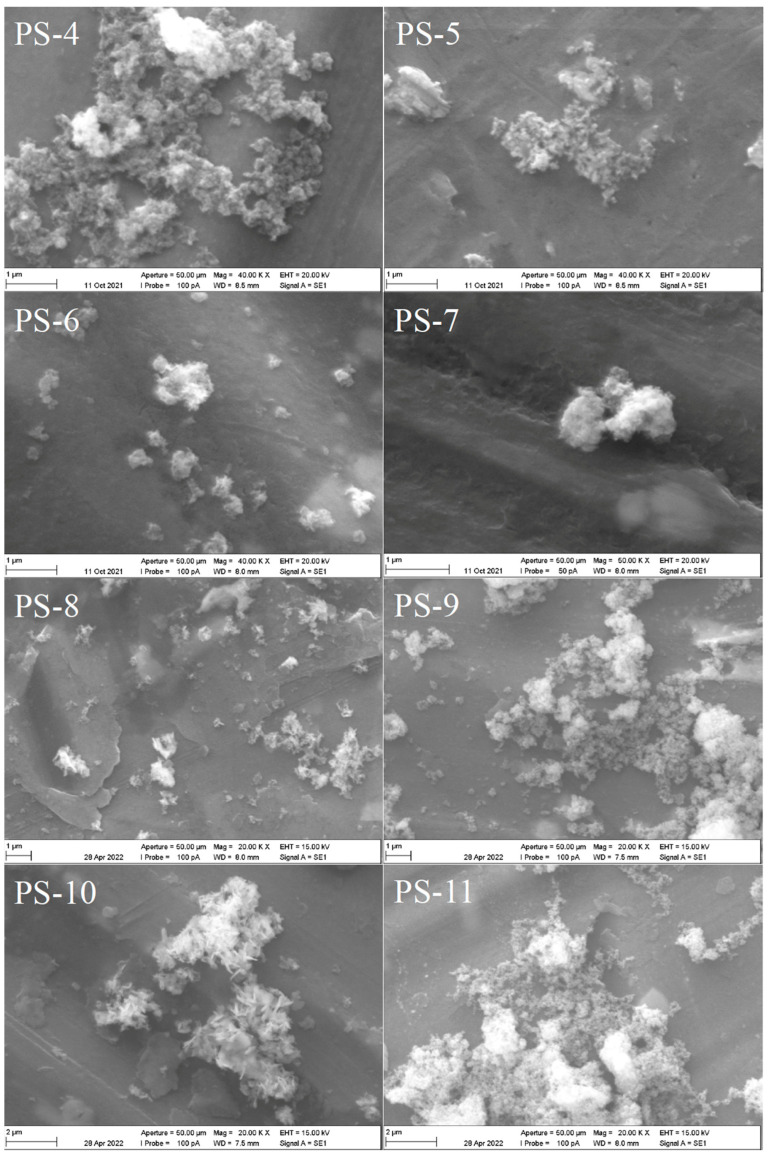
SEM images of powders obtained through precipitation stripping from experiments PS-4 to PS-11.

The remarkable broadness of the reflections gives a hint of the nanocrystalline state and the poor crystallinity, which is confirmed by the scanning electron micrographs (Figure 4).

**Figure 5:**
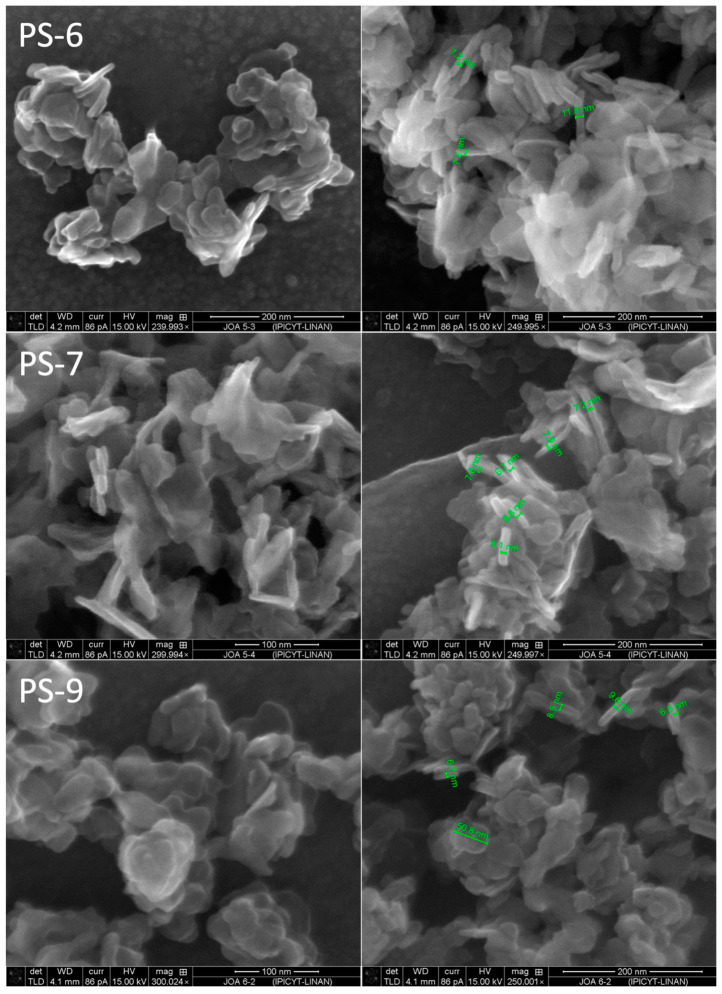
FESEM of samples PS-6, PS-7 and PS-9 (0.05 M Cu(II); 4, 2 and 2 M NaCl; 0, 20 and 20 g/L PVP; O/A: 1/1, 1/1 and 1/2).

SE micrographs of the samples of Set 2 (Figure 4) show for PS-6, PS-8 and PS-10 the typical flake-like morphology observed for the first three samples (Figure 2); the particle size is bigger for sample PS-10. For the other samples, the microscope resolution does not allow us to determine the size and morphology of the samples, but they are smaller and look like aggregated polyhedral; for this reason, selected samples were microscopically characterized using high resolution (particularly those where the recovery of vanadium as a solid was higher) and the images are presented in Figure 5.

**Figure 6:**
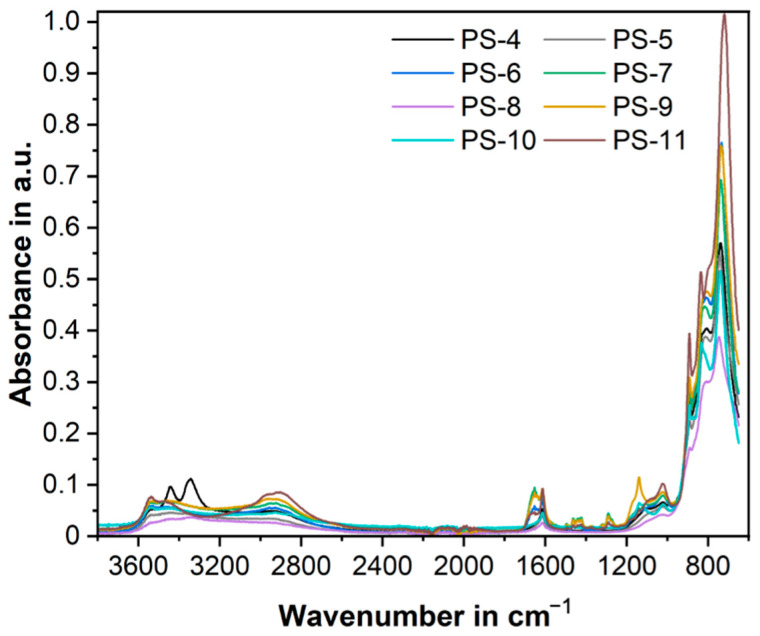
FTIR spectra of copper vanadate powders (Set 2) prepared by precipitation stripping from Aliquat^®^336 organic solutions, some of them in the presence of PVP.

The presence of PVP seems to be leading to higher recoveries of V(V) as a solid and also to smaller particle sizes. The presence of PVP on the samples was identified using FTIR, where the presence of the characteristic peaks for PVP was evident (Figure 6); for example, the weak peaks at 1653 cm^−1^ correspond to the -OH asymmetric stretching. Again, no signals corresponding to Aliquat^®^336 were found in the spectra.

As for the crystallography of volborthite (Cu_3_V_2_O_7_(OH)_2_·2H_2_O, monoclinic or pseudohexagonal structure) reported in the literature, the mineral forms rosette-like aggregates of scaly crystals [47] and is an uncommon secondary mineral, formed in the oxidized part of vanadium-bearing hydrothermal mineral deposits [48]. The crystal structures of volborthite and its synthetic analog have been reported [34,35], where the basic structure of monoclinic volborthite is considered to be a sheet-like structure with copper oxide/hydroxide layers (the copper layer, in octahedral coordination with oxygen [38]), stacked within water layers held together by means of pyrovanadate O_3_V–O–VO_3_ groups [47]. Among transition metal vanadates, Cu_3_(V_2_O_7_)(OH)_2_·2H_2_O has been studied as a flame retardant and also as an electrode for primary lithium batteries due to its high storage capacity, low cost and environmental friendliness [4,21,23]. However, drawbacks of Cu_3_(V_2_O_7_)(OH)_2_·2H_2_O as an active material in rechargeable lithium-ion batteries are the poor electrical conductivity as well as the large volume expansion during the repeated lithium cycling processes [4]. Volborthite has been also targeted as a model compound for the spin-1/2 kagome antiferromagnet [49].

The structure evolution of the flake-assembled nanoflowers is proposed to be as follows: the high chloride concentration forces the vanadate ions out of the organic and into the aqueous phase, regenerating part of the extractant, but with some copper chlorocomplexes being extracted into the organic at the same time. With the copper ions in the aqueous phase, the stripped vanadate moieties form, due to the low solubility product of copper vanadates, a large number of tiny nuclei. A lot of metal vanadates, among them Cu_3_V_2_O_7_(OH)_2_·2H_2_O, have a layered crystal structure that leads to anisotropic growth and to the formation of nanoflakes. The formed flakes self-assemble into nanoflowers in order to minimize the surface energy [50]. As the surface of the obtained sheets is quite smooth, an Ostwald ripening mechanism could be proposed for Sample PS-1. For the materials prepared in the presence of PVP, this stabilizer seems to have an important role in forcing the vanadium ions out of the organic phase and the synthesis process seems to become more efficient. The presence of PVP on the surface of the particles and the varied sizes and morphologies observed give a hint of the role of PVP as a growth controller.

Our data were not sufficient enough to confirm the crystal structure of Cu_3_V_2_O_7_(OH)_2_·2H_2_O, which, according to the literature, consists of CuO_4_(OH)_2_ octahedral units, edge-shared with each other to form Cu_3_O_6_(OH)_2_ layers, separated by V_2_O_7_ pillars and non-ligated water molecules [49], resulting in a layered structure potentially capable of allowing facile alkaline ions insertion and extraction in batteries, a subject of research in the near future.

## 4. Conclusions

In summary, copper vanadate particles have been synthesized via a facile route involving solvent extraction and precipitation stripping, where the extractant Aliquat 336 acts to control the release rate of the V(V) ions more than as a surfactant or control growth reagent. The stabilizing polymer polyvinylpyrrolidone plays a more important role in displacing the precipitation equilibrium to the formation of the metal vanadate.

The facile method, involving the selective solvent extraction process, might provide new possibilities for preparing other kind of single-phase low-solubility metal vanadate materials with different structures. We were able to identify ultrafine flakes of Cu_3_V_2_O_7_(OH)_2_·2H_2_O.

As an extension of this work, the electrochemical properties of the Cu_3_V_2_O_7_(OH)_2_·2H_2_O nanostructures as electrode materials in rechargeable batteries would be of particular interest, mainly the study of the Li^+^ intercalation and deintercalation process into the nanostructures and the cycling reversibility.

## Figures and Tables

**Table 1:** Composition of the solutions and phase ratio used in the strip and precipitation experiments.

Experiment	Cu(II), M	Cl^−^, M	PVP, g/L	O/A
	Set 1			
PS-1	0.05	4	0	1/1
PS-2	0.1	4	0	1/1
PS-3	0.05	4	20	1/1
CP-1 *	0.1	4	20	-
	Set 2			
PS-4	0.1	4	0	1/1
PS-5	0.05	4	0	1/1
PS-6	0.05	2	20	1/1
PS-7	0.05	2	40	1/1
PS-8	0.05	4	0	1/2
PS-9	0.05	2	20	1/2
PS-10	0.05	4	0	2/1
PS-11	0.05	2	20	2/1

* CP-1 conventional precipitation, phase ratio 1/1.

**Table 2:** Rietveld refinement of samples PS-1 and PS-2.

Sample	PS-1	PS-2	
Compound	Tricopper divanadate dihydroxide dihydrate Volborthite [34]	Tricopper divanadate dihydroxide dihydrate Volborthite [34]	Tricopper divanadate dihydroxide dihydrate Volborthite [35]
Molecular formula	Cu_3_V_2_O_7_(OH)_2_·2(H_2_O)	Cu_3_V_2_O_7_(OH)_2_·2(H_2_O)	Cu_3_V_2_O_7_(OH)_2_·2(H_2_O)
Molecular weight (g/mol)	474.56	474.56	474.56
wt. (%)	100	57.69	42.30
Symmetry	Monoclinic	Monoclinic	Monoclinic
Space group (H. M.)	*C*2/*m*	*C*2/*m*	*C*2/*c*
*a* (Å)	10.610(3)	10.716(7)	10.947(3)
*b* (Å)	5.912(1)	5.837(5)	6.023(2)
*c* (Å)	7.242(1)	7.160(3)	13.683(5)
*β* (°)	94.23(2)	93.78(6)	94.01(3)
Crystallite size (Å)	99.6(5)	105(1)	151(3)
*ρ*_X-ray_ (g/cm^3^)	3.47	3.52	3.47
R_wp_ (%)	1.561	1.001	
R_b_ (%)	1.112	0.706	
R_exp_ (%)	0.540	0.405	

**Table 3:** Distribution (%) of V(V) between the organic, the solid and the aqueous phases for the precipitation stripping experiments PS-1 to PS-3 and chemical composition of the obtained powders (Set 1). Addition time: 10 min.

					Distribution of V(V), %			Chemical Composition, %		V/Cu Ratio
	Cu(II) mol/L	Cl^−^ mol/L	PVP g/L	O/A	V(V) Org	V(V) Solid	V(V) Aq	V(V)	Cu(II)	
PS-1	0.05	4	0	1/1	71.7	28.3	0.0	18	42	0.43
PS-2	0.1	4	0	1/1	87.5	12.5	0.0	13.6	44	0.31
PS-3	0.05	4	20	1/1	33.4	66.0	0.6	24.8	54.4	0.45

**Table 4:** Distribution of V(V) and Cu(II) between the organic, the solid and the aqueous phases for the different precipitation stripping experiments (Set 2). Addition time: 1 h, mixing time after addition: 2 h.

	Cu(II) mol/L	Cl^−^ mol/L	PVP g/L	O/A	% V Org	% V Solid	% V Aq	% Cu Org	% Cu Solid	% Cu Aq	pH eq Aq
PS-4	0.1	4	0	1/1	61.2	38.7	0.1	43.6	19.5	36.9	3.39
PS-5	0.05	4	0	1/1	60.9	38.9	0.2	78.1	13.1	8.7	3.54
PS-6	0.05	2	20	1/1	37.1	62.8	0.1	55.8	21.0	23.2	3.84
PS-7	0.05	2	40	1/1	38.4	61.5	0.1	52.3	24.4	23.3	3.89
PS-8	0.05	4	0	1/2	68.8	31.0	0.3	72.1	4.1	23.8	3.41
PS-9	0.05	2	20	1/2	32.8	66.8	0.4	51.2	9.8	39.0	3.61
PS-10	0.05	4	0	2/1	70.9	23.8	5.3	85.6	14.2	0.1	4.82
PS-11	0.05	2	20	2/1	30.9	68.9	0.2	56.2	37.2	6.6	3.73

**Table 5:** Vanadium, copper and chloride ions in the solid (%) and V/Cu ratio in the powders obtained during the precipitation stripping experiments (Set 2).

	Cu(II) mol/L	Cl^−^ mol/L	PVP g/L	O/A	% V	% Cu	% Cl^−^	V/Cu
PS-4	0.1	4	0	1/1	13.2	33.9	4.7	0.39
PS-5	0.05	4	0	1/1	17.8	30.5	5.4	0.58
PS-6	0.05	2	20	1/1	18.5	31.5	3.3	0.59
PS-7	0.05	2	40	1/1	18.1	36.3	6.3	0.50
PS-8	0.05	4	0	1/2	17.1	22.8	3.2	0.75
PS-9	0.05	2	20	1/2	19.3	28.2	3.2	0.68
PS-10	0.05	4	0	2/1	10.4	15.5	25.1	0.67
PS-11	0.05	2	20	2/1	25.5	34.3	1.7	0.74

## Data Availability

Not applicable.

## Supplementary Materials

The following supporting information can be downloaded at: https://www.mdpi.com/article/10.3390/nano13131977/s1, Table S1: Mineralogical phases identified for the samples PS-4 to PS-11 using the software Profex. Quantification should be carefully taken into account due to the broad signal obtained for volborthite. Figure S1: X-ray diffraction pattern of Sample PS-1, copper vanadate, Cu_3_V_2_O_7_(OH)_2_·2H_2_O, strip solution 0.05 M CuSO_4_ in 4 M NaCl. Figure S2: EDS pattern and chemical analysis (inset) of Sample PS-3. Figure S3: IR spectra of Aliquat 336 and PVP. Figure S4: IR spectra of copper vanadate powders PS-1 and PS-3 prepared by precipitation stripping from Aliquat 336 organic solutions, PS-3 prepared in the presence of PVP.
