# Supplementary material for: Preparation of Volborthite by a Facile Synthetic Chemical Solvent Extraction Method
Source: Nanomaterials (Basel). 2023 Jun 29;13(13):1977. doi: 10.3390/nano13131977 (PMC10343262; doi:10.3390/nano13131977)
Supplement: Supplementary file 1 [file nanomaterials-13-01977-s001.zip › nanomaterials-2435236-supplementary.pdf]

Electronic Supporting Information (ESI)

Preparation of Volborthite by a Facile Synthetic Chemical Solvent Extraction Method

María Guadalupe Sánchez-Loredo [1,2,*], Salvador Antonio Palomares-Sánchez [3], Gladis Judith Labrada-Delgado [4], Toni Helbig [1], Paul Chekhonin [5], Doreen Ebert [1], Robert Möckel [1], Jones Owusu Afriyie [1] and Norman Kelly [1]

[1] Helmholtz-Zentrum Dresden-Rossendorf e.V., Helmholtz-Institut Freiberg für Ressourcentechnologie, Chemnitzer Str. 40, 09599 Freiberg, Germany; tonihelbig@yahoo.de (T.H.); d.ebert@hzdr.de (D.E.); r.moeckel@hzdr.de (R.M.); ojoovia@gmail.com (J.O.A.); n.kelly@hzdr.de (N.K.)

[2] Instituto de Metalurgia, Facultad de Ingeniería, Universidad Autónoma de San Luis Potosí, Sierra Leona 550, San Luis Potosí 78210, Mexico

[3] Facultad de Ciencias, Universidad Autónoma de San Luis Potosí, Av. Chapultepec 1570, San Luis Potosí 78295, Mexico; sapasa04@fciencias.uaslp.mx

[4] Instituto Potosino de Investigación Científica y Tecnológica, Camino a la Presa San José 2055, San Luis Potosí 78216, Mexico; gladis.labrada@ipicyt.edu.mx

[5] Helmholtz-Zentrum Dresden-Rossendorf e.V., Institut für Ressourcenökologie, Bautzner Landstraße 400, 01328 Dresden, Germany; p.chekhonin@hzdr.de

* Correspondence: msanchez@uaslp.mx

Table S1. Mineralogical phases identified for the samples PS-4 to PS-11 using the software Profex. Quantification should be carefully taken into account due to the broad signal obtained for volborthite

Sample	Phases	Observations
PS-4	$Cu_3V_2O_7(OH)_2.2(H_2O)$, Volborthite (ICSD_63282, Monoclinic, C12/m1), 90%. Sodium chloride 6%, Clinoatacamite 4%	Broad peaks corresponding to Volborthite and Clinoatacamite
PS-5	$Cu_3V_2O_7(OH)_2.2(H_2O)$, Volborthite (ICSD_63282, Monoclinic, C12/m1), 99.5%. Sodium chloride 0.5%	Broad peaks corresponding to Volborthite
PS-6	$Cu_3V_2O_7(OH)_2.2(H_2O)$, Volborthite (ICSD_63282, Monoclinic, C12/m1), 99%. Sodium chloride 1%	Broad peaks corresponding to Volborthite
PS-7	$Cu_3V_2O_7(OH)_2.2(H_2O)$, Volborthite (ICSD_63282, Monoclinic, C12/m1), 96%. Sodium chloride 4%	Broad peaks corresponding to Volborthite
PS-8	$Cu_3V_2O_7(OH)_2.2(H_2O)$, Volborthite (ICSD_63282, Monoclinic, C12/m1), 100%	Broad peaks
PS-9	$Cu_3V_2O_7(OH)_2.2(H_2O)$, Volborthite (ICSD_63282, Monoclinic, C12/m1), 99%. Sodium chloride 1%	Broad peaks corresponding to Volborthite
PS-10	$Cu_3V_2O_7(OH)_2.2(H_2O)$, Volborthite (ICSD_63282, Monoclinic, C12/m1), 2%. Sodium chloride 98%	Sharp peaks corresponding to NaCl
PS-11	$Cu_3V_2O_7(OH)_2.2(H_2O)$, Volborthite (ICSD_63282, Monoclinic, C12/m1), 99%. Sodium chloride 1%	

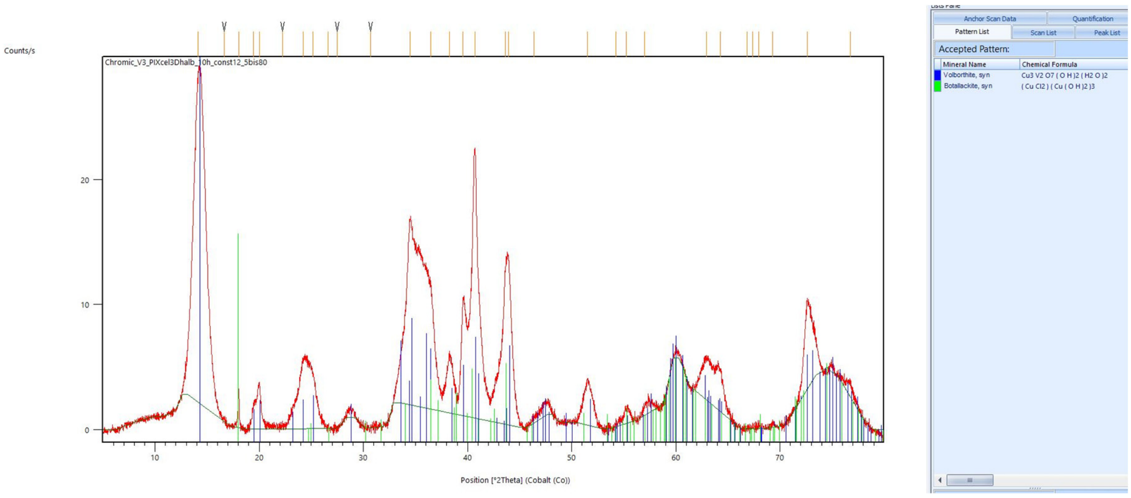

Figure S1. X-Ray diffraction pattern of Sample PS-1, copper vanadate, $Cu_3V_2O_7(OH)_2.2H_2O$, strip solution 0.05 M CuSO4 in 4 M NaCl

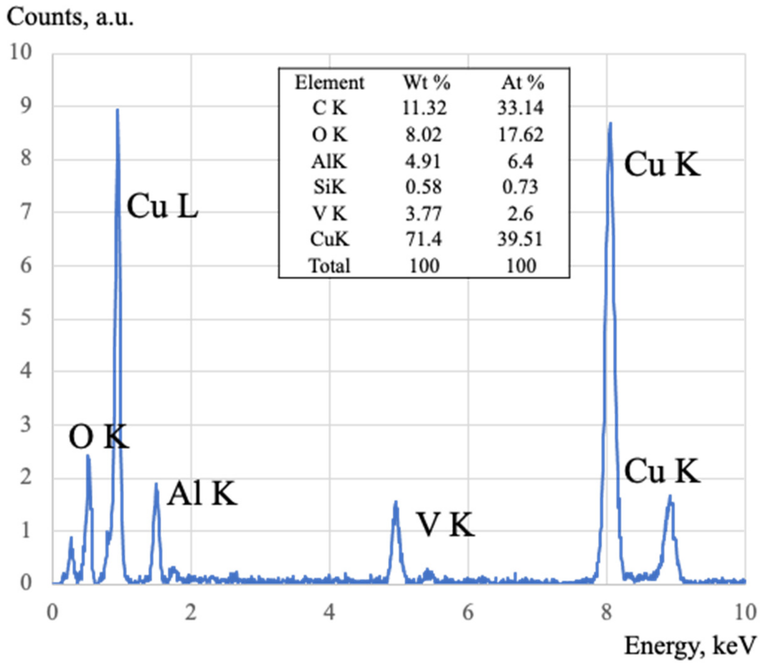

Element	Wt %	At %
C K	11.32	33.14
O K	8.02	17.62
AlK	4.91	6.4
SiK	0.58	0.73
V K	3.77	2.6
CuK	71.4	39.51
Total	100	100

Figure S2. EDS pattern and chemical analysis (inset) of Sample PS-3

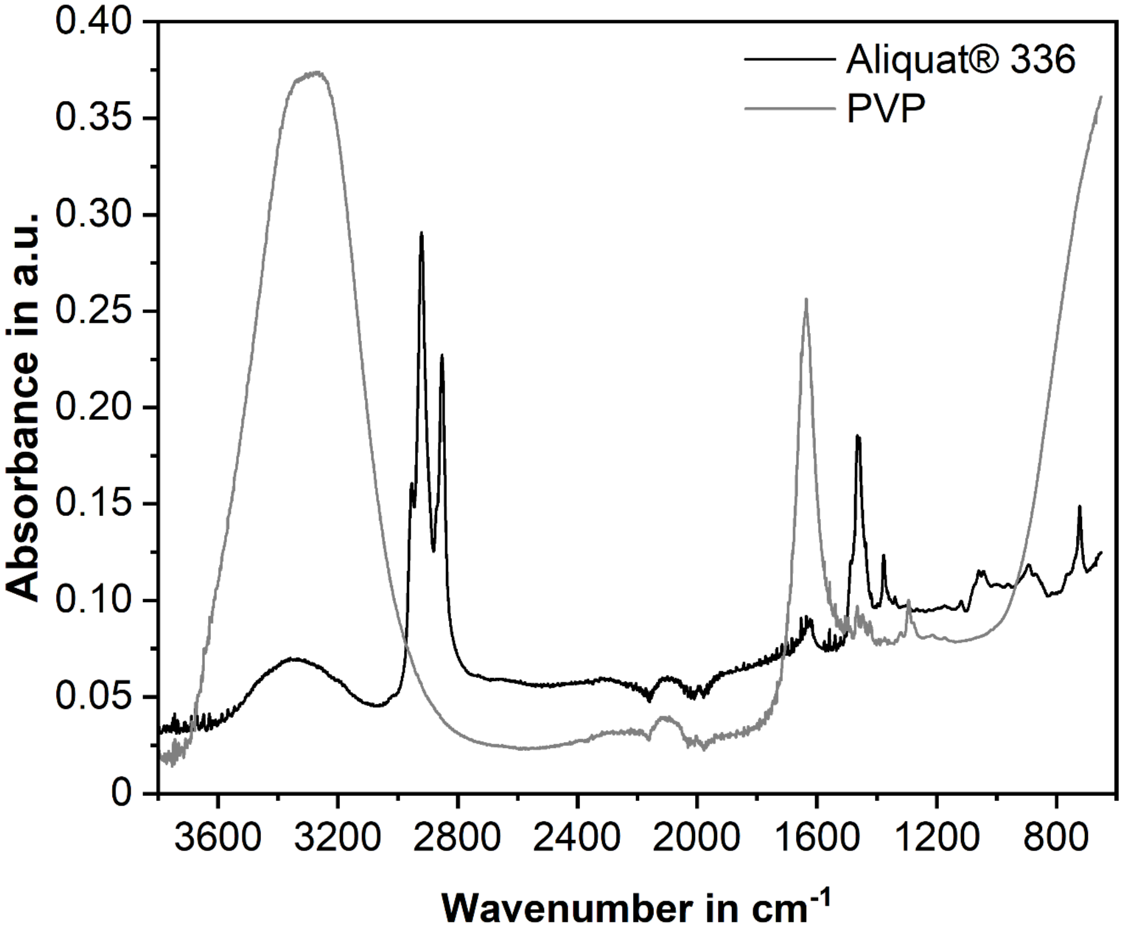

Figure S3. IR spectra of Aliquat 336 and PVP

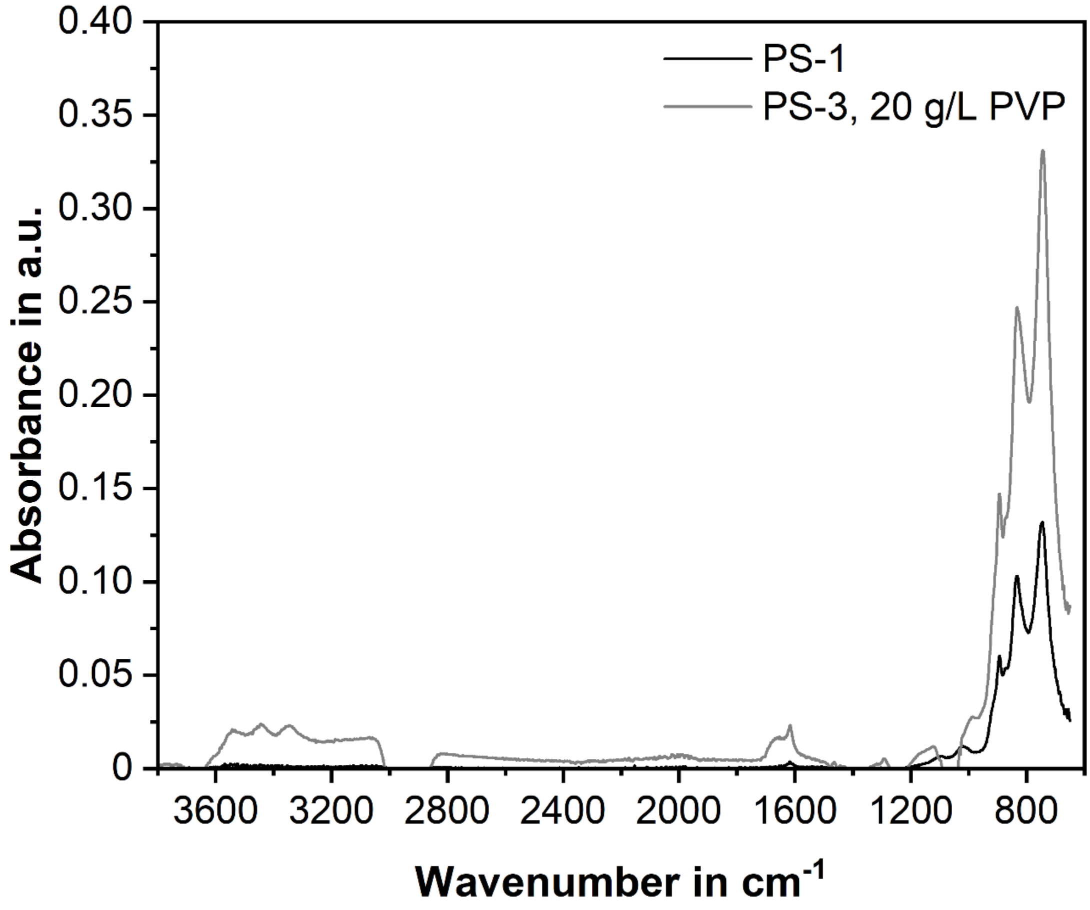

Figure S4. IR spectra of copper vanadate powders PS-1 and PS-3 prepared by precipitation stripping from Aliquat 336 organic solutions, PS-3 prepared in the presence of PVP
